# Hemp Hay (*Cannabis sativa* L.) in Grazing Goats’ Diet: Effects on Oxidative and Inflammatory Status

**DOI:** 10.3390/ani15030364

**Published:** 2025-01-27

**Authors:** Fabio Zicarelli, Daria Lotito, Piera Iommelli, Ruggero Amato, Tiziana Maria Mahayri, Nadia Musco, Eleonora Pacifico, Federico Infascelli, Raffaella Tudisco, Giuseppe Moniello, Pietro Lombardi

**Affiliations:** 1 Department of Veterinary Medicine and Animal Production, University of Napoli Federico II, 80100 Napoli, Italy; fabiozicarelli@gmail.com (F.Z.); daria.lotito@unina.it (D.L.); ru.amato@outlook.com (R.A.); nadia.musco@unina.it (N.M.); pacificoeleonora7465@gmail.com (E.P.); infascel@unina.it (F.I.); tudisco@unina.it (R.T.); pilombar@unina.it (P.L.); 2 Department of Veterinary Medicine, University of Sassari, 07100 Sassari, Italy; t.mahayri@studenti.uniss.it (T.M.M.); moniello@uniss.it (G.M.)

**Keywords:** grazing goat, health status, hemp hay, oxidative stress

## Abstract

Hemp (*Cannabis sativa* L.) is a versatile and multipurpose crop with a wide range of industrial, commercial, environmental and medicinal applications. This study aimed to evaluate the effects of hemp hay on the oxidative and inflammatory status of grazing goats. The high content of polyunsaturated fatty acids with anti-inflammatory activity makes hemp hay a promising feed that can be used to improve animals’ health by means of its metabolic profile, as well as its oxidative and inflammatory status. Studies on hemp hay suggest that its usage could replace alfalfa hay to obtain beneficial effects in terms of the milk yield and health status of goats. Indeed, because of the various biological compounds in hemp hay, the effects of its administration should be accurately explored. A total of 20 multiparous goats were equally divided immediately after calving (February) into two groups (C: control vs. H: hemp) that were homogeneous in terms of live weight (49 ± 2 kg) and milk yield at the previous lactation (2250 ± 200 g/head/day). The animals remained in the pasture but received 500 g/head/day of a barley and corn meal mixture. In addition, group H received 250 g/head/day of hemp hay, and group C was fed the same amount of alfalfa hay. Blood samples were taken before the trial and four months later and analyzed for clinical biochemistry, oxidative status, and anti-inflammatory profile. Reactive oxygen metabolites, biological antioxidant potential, Interleukins 6 (IL-6) and 10 (IL-10), and TNF-α were determined. In group H, a significant decrease in TNF-α level (22.7 vs. 12.7 pg/mL, *p* = 0.01) and an opposite but not significant trend for IL-10 was detected. In addition, there was a significant decrease (184.6 vs. 111.4 UCARR, *p* = 0.001) in d-ROM content and an increase in the biological antioxidant potential (BAP) values (3238.1 vs. 3886 μmol/L). A significant anti-inflammatory effect was only registered for TNF-α, whereas IL6 and IL10 were not affected.

## Simple Summary

Hemp (*Cannabis sativa* L.) seeds and oil have been used for various purposes, including as a feed source for ruminants. The results of this study suggest that hemp hay may be used as feed, given its high concentration of phenolic compounds and polyunsaturated fatty acids, rather than for its nutrient profile since it exhibits health-promoting qualities. The antioxidant and anti-inflammatory effects of including hemp hay in grazing goats’ diet have been shown as an improvement in the antioxidant barrier, with a consequent reduction in free radicals, and through a decrease in TNF-α, known as a pro-inflammatory cytokine.

## 1. Introduction

Hemp (*Cannabis sativa* L.) is a versatile and multipurpose crop with a wide range of industrial (from textiles to supercapacitors), commercial, environmental and medicinal applications [1,2,3,4,5]. Because of the increasing demand for animal products, new livestock production systems that benefit food security and sustainability are needed. Livestock breeding is important for rural and pastoral economies; thus, the use of local breeds is important to sustainable agriculture, mainly because of their high adaptation to specific environmental conditions and to the related traditional products. Moreover, a typical breeding system of a territory is able to characterize the productions that take place within its borders. The use of local feeding resources in a livestock production system is important to reduce the marked feed deficit and allow for the exploitation of areas that are marginal for cultivation but well suited for grazing. This is of particular interest for husbandry traditionally based on small herds of local breeds, as in Mediterranean areas, where small autochthonous ruminants are adapted to marginal pedoclimatic areas in comparison to selected breeds. The use of pasture in the Mediterranean area is of great benefit to livestock farms since it allows for a reduction in the economic inputs related to animal feeding and anchors the population to rural areas otherwise abandoned. At the same time, local feeding resources can positively influence the chemical composition and the nutritional profile of animal products, improving the functional compound’s content [6,7]. Hemp cultivation can contribute to sustainable agriculture, economic development, and environmental protection, according to the objectives of the European Green Deal [8]. Seeds are used as food for humans, whether they be raw, toasted, or ground into flour [9,10,11,12], and to produce rich omega-3 and omega-6 oils with beneficial properties that contribute to preventing cardiovascular diseases [13]. The seed meal that is a by-product of hemp oil has been reported to be a useful feed source for livestock [14]. The stalks could be used for fiber production or building materials [15], being rich in strong and durable fibers, known as hemp fiber or hemp bast fiber [16,17,18]. Hemp leaves and flowers contain other beneficial compounds, such as terpenes and flavonoids, which contribute to the plant’s potential therapeutic effects [19,20]. Hemp roots can help to improve soil quality and the recovery of contaminated sites by absorbing heavy metals and toxins [3,21]. Moreover, they may be used as biomass for energy purposes [22,23].

Hemp hay is an advantageous by-product of the supply chain, benefitting the eco-sustainability and optimization of production [24]. According to the European Commission, “hemp production will increase from 97,130 tonnes in 2015 to 179,020 tonnes in 2022 (an increase of 84.3%)” [8], so finding alternatives that use hemp production by-products is essential to guarantee the sustainability of the hemp industry. Beyond seeds, the use of hemp hay in animal feed may offer multiple advantages, including resource efficiency, cost savings and sustainability [25,26]. In addition, hemp hay may increase animal health status due to the number of bioactive compounds that it contains, mainly terpenes [23], and it may be used as feed and in bio-preservatives [24], representing an advantage for the agri-food industry. The incorporation of such products into animal feed contributes to a circular economy, where resources are used efficiently, and waste is minimized [27].

Studies on hemp hay have suggested that its usage could replace alfalfa hay to obtain beneficial effects in terms of the milk yield [28] and health status of goats [29]. Indeed, because of the various biological compounds of hemp hay, the effects of its administration should be explored. Of these, terpenes are generally considered beneficial but have also been reported to negatively affect oxidative status [30].

Thus, the aim of this study was to explore the effects of hemp hay in the diets of grazing goats on clinical biochemistry, oxidative stress, and inflammatory status. The hypothesis was that the inclusion of hemp hay in goat rations can provide a balanced diet for animals, which may enhance animal health by enhancing oxidative and inflammatory status.

## 2. Materials and Methods

### 2.1. Animals and Diet

Twenty multiparous lactating goats (breed: Murciana; calving: third) from a farm located in Salerno province (Italy) (12 ha; 832 m.s.l.; 40°09′ N, 15°37′ E; 30–68 mm of rainfall; temperature of 9.8–22 °C) were equally divided at 60 ± 7 days in milk into two groups (C—control vs. H—hemp) homogeneous in terms of body weight 49 kg (±2.0 kg), parity (3rd), and milk yield (2250 ± 200 g/head/day). The animals were milked twice per day for 4 months (from April to July). Both groups were grazing in a permanent pasture—9:00 a.m. to 4:00 p.m.—composed of forage (*Poaceae*, *Fabaceae*, *Asteraceae*), shrubs (*Erica* spp.; *Arbutus* spp.; *Prunus spinosa*) and trees (*Quercus ilex* and *Quercus pubescens*) and had free access to water.

When not grazing, each goat was housed in a 1 × 2 m box and fed 500 g/head/day of barley and corn meal (AH); additionally, group H was administered 250 g/head/day of hemp hay (HH), whereas group C received the same amount of alfalfa hay. Individual dry matter (DM) intake was measured by subtracting eventual refusals from administered feed. The pasture intake was estimated to be 980 g DM, considering that the average pasture DM intake in the internal areas of southern Italy is 20 g/kg BW [31]. The chemical composition and nutritive value of the administered feeds are reported in Table 1.

With the aim of reducing the differences in chemical composition observed in previous studies [32], pasture was gathered every 7 days from four areas of 2.5 m^2^, dried at 65 °C and pooled monthly. Pasture, hemp and alfalfa hay, barley and corn samples were milled through a 1 mm screen and analyzed in accordance with the AOAC [33] for dry matter (DM, ID 934.01), crude protein (CP, ID 984.13), ether extract (EE, ID 920.29) and ash (ID: 942.05). The fiber fractions were evaluated as described by Van Soest et al. [34], and the nutritive value (UFL = 1700 kcal of net energy for lactation) was calculated [35].

### 2.2. Blood Collection and Serum Biochemistry

All procedures were carried out at the Department of Veterinary Medicine and Animal Production (University of Napoli Federico II) after approval by the Bioethics Committee (protocol number: PG/2019/0070006). Before the onset of the experiment and after 4 months with different hay supplementations, 5 mL of blood was collected after 12 h of fasting via jugular venipuncture from each goat into vacutainer plastic tubes (Becton Dickinson, NJ, USA) and centrifuged (1500× *g* for 15 min) to obtain serum. Serum samples were stored at −80 °C and defrosted immediately before the serological, biochemical, and oxidative stress and inflammation marker assays. Blood chemistry analyses were performed on serum aliquots using reagents from Spinreact (17176 Sant’Esteve de Bas, Girona, Spain) following the manufacturer’s instruction to determine liver (total proteins, albumin, aspartate aminotransferase and total bilirubin) and kidney indicators (blood urea nitrogen and creatinine) via an automatic biochemical analyzer (AMS Autolab, Diamond Diagnostics, Holliston, MA, USA).

### 2.3. Measurements of Oxidative Stress and Inflammation Markers

Reactive oxygen metabolite-derived compounds (d-ROMs) and biological antioxidant potential (BAP) were assayed by use of an automatic biochemical analyzer (AMS Autolab, Diamond Diagnostics, Holliston, MA, USA) using reagents from Diacron (Diacron International SRL, Grosseto, Italy). Concerning the d-ROM test, because of the Fenton reaction, when iron is released by plasma proteins in an acidic buffer, reactive oxygen metabolites generate alkoxyl and peroxyl radicals. Then, these radicals oxidize an alkyl-substituted aromatic amine (N, N-dietyl-paraphenylendiamine), producing a pink derivative that can be quantified at 505 nm. The d-ROM level, proportional to the color intensity, is expressed in Carratelli Units (1 CARR U = 0.08 mg hydrogen peroxide/dL). In the BAP test, by adding a plasma sample to a colored solution produced by mixing ferric chloride and thiocyanate derivative, a discoloration is obtained, and its intensity, measured at 505 nm, is proportionate to the plasma-induced ferric ion reduction. The results are expressed as mol/L of reduced ferric ions [36].

Serum tumor necrosis factor-alfa (TNF-α), interleukin 6 (IL-6) and interleukin 10 (IL-10) were measured using goat ELISA kits (Genorise, Glen Mills, PA, USA) following the manufacturer’s instructions. The detection range assay for TNF-α was 1–2200 pg/mL, with the CV intra- and inter-assay values of <7% and <9%, respectively. The IL-6 detection range was 50–3200 pg/mL with the intra- and inter-assay CV values < 6% and <9%, respectively. The IL-10 detection range assay was 25–1600 pg/mL with the intra- and inter-assay CV values of <5% and <8%. Absorbance was determined using an automated microplate spectrophotometer (Epoch, BioTek Instruments Inc., Winooski, VT, USA) at 450 nm.

### 2.4. Statistical Analysis

Data were analyzed using the two-way ANOVA procedure of JMP^®^ (version 14; SAS Institute, Cary, NC, USA), according to the following model:

Yijk = m + Gi + Sj + (DS)ij + εijk

where Yijk = single observation; m = general mean; Gi = group effect (i = C and H); Sj = sampling effect (j = 1, 2); DS = interaction between the group and sampling effect; and εijk = the residual error. The means were statistically compared using Tukey’s test. Differences were considered statistically significant at *p* < 0.05.

## 3. Results

The feeds’ chemical compositions are reported in Table 1. No refusals of the barley and corn meal, hemp hay or alfalfa hay were detected for either group. The protein content and energy value of the pasture were similar to those reported in previous studies in the same area [37,38]. The nutritive value of HH was similar to that of AH (UFL/kg DM 0.78 vs. 0.77, for HH and AH, respectively), as were the crude protein contents (207.7 ± 2.7 vs. 194.5 ± 2.6 for HH and AH, respectively).

As reported in Table 2, no significant differences were observed between the two groups in terms of blood biochemistry parameters. Despite this, urea showed an opposing trend (*p* = 0.617) between the two groups, increasing in group C (19.33 ± 3.4 vs. 23.83 ± 2.9 mg/dL, +23.2%) and decreasing in group H (23.17 ± 3.1 vs. 21.67 ± 2.7, 6.5%) at the end of the trial.

For both oxidative and inflammatory markers, while no difference was detected in the control group after four months of trial, TNF-α, d-ROMs and BAP changes occurred in the group fed hemp hay. The oxidative status (Table 3) test revealed that the reactive oxygen metabolites (d-ROMs) were significantly lower (*p* < 0.01) in the experimental group after the hemp hay treatment. Additionally, an improvement in the biological antioxidant potential, as confirmed by the significantly (*p* < 0.05) higher levels of BAP, was detected in group H after the hemp hay treatment. In group H, a significant (*p* < 0.05) reduction in TNF-α was found after treatment with hemp hay, while an opposite but not significant trend was observed for IL-10. No differences were detected for IL-6. A significant interaction (*p* < 0.01) between group and time was registered for d-ROMs, BAP and TNF-α.

## 4. Discussion

Clinical biochemistry tests showed that hemp hay was well tolerated [39]. The absence of adverse effects, at least with regard to biochemical and oxidative markers, is often a critical point in the management of animal feeding when using both drugs and nutraceuticals. Despite the absence of a significant difference in serum urea concentration, an opposite trend was registered between the two groups, showing a decrease in the treated group, according to Irawan et al. [40], but an increase in the control group. Indeed, all levels fall within the normal range for goats.

Cytokines comprise the main regulatory system at play during inflammation and host defense against bacterial infections. They can be involved in the immunopathologic process and are considered diagnostic markers in several diseases [41]. In this study, a significant (*p* < 0.05) decrease in TNF-α and an opposite but non-significant trend for IL-10 was detected in goats supplemented with hemp hay. The significant (*p* < 0.05) reduction in TNF-α after treatment suggests that hemp administration was active in modulating cytokine levels compared to the standard diet [41].

TNF-α is a pro-inflammatory cytokine produced by activated macrophages, T lymphocytes and natural killer (NK) cells [42]. In group H, TNF-α was significantly lower (*p* < 0.05) after four months, thus supporting the hypothesis that hemp administration could be able to prevent the negative effects of the pro-inflammatory pathways that can occur mainly during the first stage of lactation [43]. Such a hypothesis seems to be confirmed by the increase (49.39 ± 23.6 vs. 58.32 ± 19.6), even if not statistically significant, in IL-10. IL-10 is a cytokine with several effects in immunoregulation and inflammation. It is known to suppress cytokine secretion, antigen presentation and CD4+ T cell activation, and in particular, it predominantly inhibits the lipopolysaccharide- (LPS) and bacterial product-mediated induction of a number of pro-inflammatory cytokines, including TNF-α [44]. Our results show significant decreases in TNF-α and IL-6, both of which are generally considered pro-inflammatory cytokines. Indeed, IL-6 has been reported to act through both pro- and anti-inflammatory mechanisms [45]. The non-significant increase in IL-10 may be due to some of the limitations of this study, such as the limited number of animals and/or the dose and duration of hemp supplementation.

Significantly lower levels of d-ROMs and higher levels of BAP were detected in group H. The d-ROM test provides a practical method for identifying high levels of oxidative stress [36] by analyzing the total amount of hydroperoxides in serum products of ROS (reactive oxygen species) [46]. This test provides an indication of the balance between oxidative stress and antioxidant defenses, helping assess the risk of diseases associated with oxidative stress [43]. Therefore, the decrease in d-ROMs in the treated group showed the positive effect of hemp hay feed supplementation; also, animals’ oxidation status could represent a potential marker of their health status [39]. Free radicals are formed from molecules via the breakage of a chemical bond such that each fragment keeps one electron via the cleavage of a radical to give another radical via redox reactions [47]. Physiological concentrations of ROS play an important role in normal cellular functions, and high concentrations cause damage at the cellular level because cellular antioxidant mechanisms fail to counteract them. Similar results of the usage of cannabis supplementation have also been observed by Wang et al. [48], who reported on the use of hempseed lignanamide to regulate the cellular antioxidant response against ROS [48].

In addition, our results show an opposing increase in BAP concentration in the group with hemp hay supplementation compared to the alfalfa hay group, confirming our hypothesis on the beneficial effects of *Cannabis sativa* L. in ruminants’ diets. The BAP test is used to provide a global measurement of non-enzymatic antioxidants [49]. In particular, the increasing BAP in group H indicates the chemically active antioxidant capacity (scavengers) of the plasma barrier. Specifically, it includes antioxidants, both exogenous (ascorbic acid, terpenes, flavonoids and tocopherols) and endogenous (uric acid, bilirubin and albumin) in nature. As reported by Hassan et al. [50], *Cannabis sativa* L. contains naturally occurring antioxidants; thus, it can be used to control oxidative stress [50]. Moreover, *Cannabis sativa* has a high number of anti-inflammatory properties and may control oxidative processes during inflammation [51]. According to previous research, our results show that group H enhanced the oxidative status with an improvement in the antioxidant barrier and a consequent reduction in free radical formation. This confirms the hypothesis of an antioxidant effect of *Cannabis sativa*. Such a result is also confirmed by the significant interaction (*p* < 0.01) between the group and time registered for d-ROMs, BAP and TNF-α. Thus, in addition to the potential anti-inflammatory effect, our results suggest that hemp hay could represent a valid healthy supplement in ruminant diets. The effects described above are likely to be attributed to the higher levels of MUFA, which could act on ruminal metabolism [28]. Indeed, when exposed to MUFA, macrophages show a lower level of inflammation. AMPK may also act in reducing inflammation by inhibiting NFκB activity, thus impeding the priming of pro-IL-1β. MUFA-treated cells showed greater anti-stress activity, reducing glycolysis and increasing oxidative phosphorylation. Overall, MUFA, via AMPK, seems to be able to both block and reverse SFA-induced inflammation.

While other studies showed that hemp seed supplementation can improve antioxidant status in rats [52,53], cows [54], goats [55] and sows [56], few studies on hemp hay usage have been conducted. The use of hemp hay as a feed supplementation in animal diets should be promoted in order to improve the general health status of animals. However, to confirm our hypothesis regarding the beneficial effects of *Cannabis sativa* in ruminants’ diets, further studies should be conducted to evaluate the use of hemp hay in confined breeding by testing different dosages.

## 5. Conclusions

The results suggest that hemp hay may represent a healthy feed in ruminants’ diets due to an improvement in the antioxidant barrier with a consequent reduction in ROS production. The significant decrease in TNF-α is suggestive of hemp’s potential beneficial effects on inflammatory status, but further studies are needed to clarify this last point since both IL-6 and IL-10 (pro- and anti-inflammatory cytokines, respectively) were not significantly affected by the dietary treatment.

## Figures and Tables

**Table 1:** Chemical composition (g/kg DM ± SD) and nutritive value (UFL/kg DM ± SD) of feeds administered to the control group (group C, fed alfalfa hay) and the experimental group (group H, fed hemp hay) before (Time 0) and after 120 days (Time 1) of the trial.

	Pasture	Barley/Corn Mix	Hemp Hay	Alfalfa Hay
Crude protein	160.2 ± 1.3	100.4 ± 0.9	207.7 ± 2.7	194.5 ± 2.6
Ether extract	22.6 ± 0.3	31.6 ± 2.6	21.2 ± 0.7	16.8 ± 0.8
NDF	481.8 ± 5.1	165.2 ± 1.3	401.5 ± 2.4	413.5 ± 2.3
ADF	429.4 ± 2.51	45.1 ± 1.7	318.4 ± 5.2	344.1 ± 4.9
ADL	46.2 ± 2.9	5.3 ± 2.2	33.4 ± 6.0	51.7 ± 2.7
Ash	72.5 ± 2.2	10.5 ± 1.5	101.9 ± 1.4	99.5 ± 1.9
UFL/kg DM	0.76	1.04	0.78	0.77

DM, dry matter; NDF, neutral detergent fiber; ADF, acid detergent fiber; ADL, acid detergent lignin; UFL, unit feed for lactation. Pasture composition: forage (*Poaceae*, *Fabaceae*, *Asteraceae*), shrubs (*Erica* spp.; *Arbutus* spp.; *Prunus spinosa*) and trees (*Quercus ilex* and *Quercus pubescens*).

**Table 2:** Blood biochemistry parameters of the control group (group C, fed alfalfa hay) and experimental group (group H, fed hemp hay) before (Time 0) and after 120 days (Time 1) of the trial.

	UREA mg/dL		CREATININE mg/dL		TOTAL PROTEIN g/dL		ALBUMIN g/dL		BILT mg/dL		AST U/L	
	GROUP C	GROUP H	GROUP C	GROUP H	GROUP C	GROUP H	GROUP C	GROUP H	GROUP C	GROUP H	GROUP C	GROUP H
	21.58	22.42	0.755	0.738	7.97	7.80	2.90	2.85	0.328	0.357	113.08	104.58
Time 0	19.33	23.17	0.753	0.725	7.79	7.84	2.88	2.86	0.327	0.330	83.67	100.33
Time 1	23.83	21.67	0.757	0.752	8.16	7.76	2.91	2.83	0.377	0.337	142.5	108.83
*p*-value Group	0.780		0.545		0.508		0.600		0.462		0.721	
*p*-value Time	0.617		0.586		0.572		0.981		0.26		0.167	
*p*-value Time × Group	0.321		0.671		0.394		0.796		0.385		0.296	
RMSE	7.226		0.066		0.619		0.241		0.061		57.45	

RMSE, root mean square error; BILT, total bilirubin; AST, aspartate aminotransferase.

**Table 3:** Oxidative and inflammation status of the control group (group C, fed alfalfa hay) and experimental group (group H, fed hemp hay) before (Time 0) and after 120 days (Time 1) of the trial.

	IL-10 pg/mL		IL-6 pg/mL		TNF-α pg/mL		d-ROM UCARR		BAP μmol/L	
	GROUP C	GROUP H	GROUP C	GROUP H	GROUP C	GROUP H	GROUP C	GROUP H	GROUP C	GROUP H
	50.4	56.7	38.6	41.5	27.5 a	15.6 b	187.3 a	136.7 b	3572	3549.1
Time 0	51.7	49.4	38.9	39.5	23.4	22.7 A	202.6	184.6 A	3792.5	3238.1 B
Time 1	52.7	58.3	22.6	41.4	22.6	12.7 B	179.8	111.4 B	3238.1	3886.4 A
*p*-value Group	0.723		0.328		0.044		0.090		0.374	
*p*-value Time	0.127		0.549		0.01		0.001		0.025	
*p*-value T × G	0.241		0.310		0.01		0.01		0.008	
RMSE	15.7		12		8.27		33.4		105.9	

RMSE, root mean square error; IL-10, interleukin 10; IL-6, interleukin 6; TNF-α, tumor necrosis factor-alpha; d-ROMs, reactive oxygen metabolites; BAP, biological antioxidant potential. Note: Within a column, means without a common letter differ (capital letters: *p* < 0.01 A–B).

## Data Availability

The data presented in this review are available from the corresponding author on request.
